# A Systematic Review on TeleMental Health in Youth Mental Health: Focus on Anxiety, Depression and Obsessive-Compulsive Disorder

**DOI:** 10.3390/medicina57080793

**Published:** 2021-07-31

**Authors:** Laura Orsolini, Simone Pompili, Virginio Salvi, Umberto Volpe

**Affiliations:** Unit of Clinical Psychiatry, Department of Clinical Neurosciences/DIMSC, School of Medicine, Polytechnic University of Marche, Via Tronto 10/A, 60126 Ancona, Italy; smn.pmpl@gmail.com (S.P.); v.salvi@staff.univpm.it (V.S.); u.volpe@staff.univpm.it (U.V.)

**Keywords:** adolescents, adolescence, affective disorders, anxiety, obsessive-compulsive disorder, TeleMental Health, telepsychiatry, youngsters, youth, youth mental health

## Abstract

*Background and Objectives*: The Internet is widely used and disseminated amongst youngsters and many web-based applications may serve to improve mental health care access, particularly in remote and distant sites or in settings where there is a shortage of mental health practitioners. However, in recent years, specific digital psychiatry interventions have been developed and implemented for special populations such as children and adolescents. *Materials and Methods*: Hereby, we describe the current state-of-the-art in the field of TMH application for young mental health, focusing on recent studies concerning anxiety, obsessive-compulsive disorder and affective disorders. *Results:* After screening and selection process, a total of 56 studies focusing on TMH applied to youth depression (*n* = 29), to only youth anxiety (*n* = 12) or mixed youth anxiety/depression (*n* = 7) and youth OCD (*n* = 8) were selected and retrieved. *Conclusions:* Telemental Health (TMH; i.e., the use of telecommunications and information technology to provide access to mental health assessment, diagnosis, intervention, consultation, supervision across distance) may offer an effective and efficacious tool to overcome many of the barriers encountering in the delivery of young mental health care.

## 1. Introduction

The dissemination of interconnected networks, the growth and spread of new communication and information technologies (IT) and the capillary digitalization across all ages at different levels, have rapidly led to an increasing usage of the Internet and online tools worldwide [1], being smartphones and other online devices used nowadays as primary means of source of information and as preferred mode for social communication and peer interaction, particularly among ‘*digital native*’ young people [1,2]. Young people born after 1993 (i.e., when the Internet became widely available) have been referred to as ‘*digital natives*’ as they fundamentally grew up with the Internet and within the digital revolution [3,4]. A systematic review evaluating the effectiveness of online services in facilitating mental health seeking care in young people aged 14–25 years, reported in 38.4% of the sample a preference in online seeking mental health information [5]. Overall, the current digitalization may effectively facilitate the delivery and increase the access to mental health care among youngsters, particularly due to the current COVID-19 pandemic [6,7,8]. Within this context, Telemental Health (TMH) was born mainly due to an increased need in offering an equity access to mental health care to those people living in rural and remote areas, reducing the logistic and financial barriers encountering by people seeking help for a mental health condition and to overcome the lack of local specialty professionals in specific mental health areas such as Child and Adolescent Psychiatry (CAP) and CAP subspecialties (i.e., Autism Spectrum Disorders, Attention-Deficit-Hyperactivity Disorders, etc.) [7,9]. Several studies reported that TMH interventions may deliver and substitute any in-person mental health care [10,11,12,13,14,15]. Moreover, mobile health (mHealth), e-Mental Health and social media have been also demonstrated to be effectively used to access and treat patients at all ages, including adolescents, their parents and families [9,15,16].

Within the context of Youth Mental Health, TMH interventions have been successfully used to provide mental health services to children, adolescents and their parents, by offering several delivering modalities (i.e., audio call or audio plus video call) and typologies of interventions (i.e., telepsychiatry, telepsychotherapy, psychoeducation, etc.) [8,9,17,18] (Table 1). TMH held the promise to reach a larger population of youngsters that relies on paediatric or primary care, school, and juvenile correctional populations for their mental health assessment and treatment [8,17]. Overall, the youngsters usually display a greater comfort and satisfaction in receiving a treatment through technology compared to earlier generations, including their parents [5,17,19,20,21].

### 1.1. TeleMental Health

TMH comprise the use of real-time and interactive synchronous videoconferencing (VC), including telepsychiatry and telepsychotherapy, synchronous audioconferencing (i.e., audio call) as well as asynchronous technology modalities, including e-mails, text-based mobile health interventions, chatbots, e-consultations, and standalone remote devices [7,18,22,23,24,25]. TMH may include the process of mental health assessment, diagnosis, online-based interventions, consultations, supervision, and information across distance [10,12,13,25]. 

### 1.2. Telepsychiatry and/or Telepsychotherapy via Audio and/or Video Calls

In 1973, the term ‘telepsychiatry’ was first used to describe consultation services provided from the Massachusetts General Hospital to a medical site in Boston’s Logan International Airport and the Bedford Veteran’s Administration [26]. Telepsychiatry consists of a synchronous (i.e., 2-way) VC service rendered via a real-time interactive audio and video telecommunication system, used to provide a psychiatric evaluation, consultation, supervision, education and treatment [25]. The real-time interaction is provided between a psychiatrist and a patient who is located at a distant site from the physician or other qualified health care professional. Telepsychiatry requires little adaptation to provide care comparable to usual in-person care, being flexible and being a reasonable alternative to office visits for patients who cannot readily access needed care [7,25]. TMH may be as well delivered only by using telephony (i.e., voice/audio calls). Voice phone calls represent the oldest form of TMH and has been used in the treatment and management of several psychiatric conditions, including anxiety disorders, suicidal ideation and depression [18,19,27]. 

### 1.3. Asynchronous Technology Modes

Apart from the conventional, real-time, interactive, bi-directional and audio-video communication, other technology modes are becoming the mainstream in mental health care, including those ‘asynchronous modalities’ such as mobile technology which are integrated in phones, wearable devices and sensors, e-mails, text-based mobile health interventions, chatbots, e-consultations, and standalone remote devices [13]. e-Mail interviewing with youngsters represents a cost-effective and flexible modality able to provide to clinicians a written transcript for analysis, allowing clinicians’ reflections and give an adequate and balanced feedback to the adolescent [13]. Text-based mobile health interventions (i.e., text messaging or short message services, SMS) have been used as an mTherapy intervention, which guarantees also the immediate delivery of interventional reminders, supportive messages, self-monitoring and informative sharing [19]. Text-based communication are fast and allow an interaction, which potentially builds youngsters’ trust [13]. Evidence-based apps, eTools and social networking may promote self-managed mental health and wellbeing for youngsters and support integrated online initiatives with schools, workplaces and universities [23]. mTherapy refers to the use of mobile phone devices, smartphones and mobile applications (aka ‘apps’) in the delivery of mental health care services. Currently, mTherapy offer help with diagnosing, self-monitoring, self-awareness, symptom tracing and documentation, appointment and therapy homework reminders as well as adherence to traditional therapy [19]. In addition, e-consultations (aka e-consult/eConsult) may involve a physician who asks for a consultation related to questions outside of their expertise by using a VC modality [10]. Finally, sensors and wearable technologies (i.e., patches, bandages, shirts, smartwatches, smart glasses, wristbands, etc.) may offer a further digital tool useful for monitoring, assessing and treating Youth Mental Health conditions [15,16,28].

### 1.4. Aims of the Paper

The current COVID-19 pandemic posed clinicians in the condition to modulate and change mental health service delivery and treatment in order to guarantee the continuity and the management of *ex novo* mental health issues. However, most countries have not adequately prepared and equipped in the implementation process of TMH, especially in the context of Youth Mental Health. The major concerns and obstacles reside in the lack of equipment for TMH as well as a general poor IT knowledge and skill among mental health professionals. However, VC or audio calls TMH interventions appear to be the most feasible and easy to access and to be administered worldwide, especially in low-middle income countries. For this reason, the aim of the present paper is at providing an overview on the current state-of-the-art in the field of TMH interventions in Youth Mental Health, by focusing on both therapist-delivered online psychotherapy and telepsychiatry by the use of VC and audio calls, by excluding all interventions based on m-Health, MHealth and smartphone systems which may not be easily accessible to all countries. 

Furthermore, being mental health problems among young people mainly represented by anxiety, depression and OCD, we conducted a retrospective file systematic review of all peer-review literature regarding the application of TMH in youth depression, anxiety and obsessive compulsive disorder. A review of all objectively selected, critically assessed, and reasonably synthetized evidence on available published data, available up to 25 January 2021, was undertaken.

## 2. Materials and Methods

### 2.1. Search Sources and Strategies

A systematic literature search, according to the Preferred Reporting Items for Systematic Review and Metanalysis (PRISMA) guidelines, was performed by using the following electronic databases (last update: 25 January 2021): PubMed, MEDLINE, EMBASE and Google Scholar online databases, by combining the search strategy of free text terms and exploded MESH headings for the topic of “*Telemental health (TMH)*”, “*Telepsychiatry*”, “*Telepsychotherapy*” and “*Adolescent Psychiatry*” as follows: (((Telepsychiatry[Title/Abstract]) OR (Telemental health[Title/Abstract]) OR (Telepsychotherapy[Title/Abstract]) OR (Tele*[Title/Abstract]) OR (remote[Title/Abstract]) OR (Videoconferencing[Title/Abstract])) AND (Youth Mental Health[Title/Abstract])). A further search strategy was applied with the following keyterms: (((Telepsychiatry[Title/Abstract]) OR (Telemental health[Title/Abstract]) OR (Telepsychotherapy[Title/Abstract]) OR (Tele*[Title/Abstract]) OR (remote[Title/Abstract]) OR (Videoconferencing[Title/Abstract])) AND ((Depress*[Title/Abstract]) OR (Anxiety[Title/Abstract]) OR (Obsessive Compulsive[Title/Abstract]))). The strategy was first developed in MEDLINE and then adapted for use in other databases (Appendix A). The search was limited to “humans”, English papers and peer-reviewed journals. No limitation on the year of publication of was applied. Full papers of all potentially relevant studies and those papers whose abstract was insufficient to determine eligibility, were obtained and analyzed. Full text papers were further screened and discarded if they did not meet the inclusion criteria. Further references were retrieved through hand-searches of reference listings of review studies and of the included studies were also checked and examined for further references to be included.

### 2.2. Study Selection, Data Extraction and Management

We evaluated all studies specifically investigating TMH in Youth Mental Health, by excluding all papers addressing only adult people, m-Health, MHealth, and applications. We examined all titles and abstracts and obtained full texts of potentially relevant papers. Duplicate publications were excluded. All abstracts and titles describing apps for mobile devices (mHealth), e-Mental Health or virtual reality exposure treatment were excluded, even though they were targeted to young people. All experimental and observational study designs were considered, excluding case reports. Randomized, controlled clinical trials involving humans were prioritized, even though observational studies have been considered and included if pertinent. Narrative and systematic reviews, letters to the editor and book chapters were excluded as well for the specific aim of the systematic review but considered useful for the background of the research. In particular, all abstracts and titles evaluating TMH in Youth Mental Health were included if they were pertinent to the treatment of depression, anxiety and/or Obsessive Compulsive Disorder (OCD). Whilst those addressed to neurodevelopmental disorders, autism spectrum disorders (*n* = 13), youth bipolar disorder (*n* = 0), youth Attention-Deficit/Hyperactivity Disorder (ADHD) (*n* = 7), youth psychosis/schizophrenia (*n* = 5), youth eating disorders (*n* = 2) and youth posttraumatic stress disorder (PTSD) or stress-related disorders (*n* = 1) were excluded. Moreover, those studies recruiting young people affected with depression, anxiety or obsessive compulsive disorders but not specifically evaluating the efficacy of TMH services were excluded in the present analysis, such as those specifically focusing on the level of satisfaction, level of engagement and users’ experiences and perceptions.

### 2.3. Characteristics of Included Studies

Of the 840 abstracts initially identified, 305 were excluded because of duplicates. Of the 534 remaining papers, 151 papers have been excluded as they do are not pertinent with the main topic of the present review (i.e., studies not addressed to TMH, studies not concerning with the application of TMH in mental disorders, etc.); whilst 63 studies were eliminated because of reviews or metanalyses, 19 are opinion papers or protocols on Youth TMH services, 6 are TMH protocols not designed to youngsters, 7 are guidelines and 16 are letters to editor. Amongst those remaining 273 papers, 8 are case reports/case series and 27 are surveys regarding the perceptions and level of satisfaction amongst clinicians who provide a TMH to youngsters or amongst young people or their parents. Further 182 articles were eliminated from the remaining 237 full-text articles after an assessment of eligibility by reading the full text articles, as they are pertinent to only TMH Adult services, without data on youngsters (*n* = 137) or they are addressed to young people but not specifically on depression, anxiety or obsessive compulsive disorder (*n* = 45). Finally, a total of 56 full-text articles met the inclusion criteria for the present review, as illustrated in the flow diagram detailing the review process and results at each stage (Appendix B). The findings have been classified according to the following diagnostic categories: studies on TMH applied to youth depression (*n* = 29), to only youth anxiety (*n* = 12) or mixed youth anxiety/depression (*n* = 7) and youth OCD (*n* = 8) and summarized in Table 2. 

## 3. TMH in Youth Mental Health

Research supported the feasibility and efficacy of computer-based treatments for youth anxiety and related disorders [29,30,31,32,78,79]. Several TMH sessions are based on self-administered platforms and behavioural intervention technologies with or without minimal therapist support, only leverage asynchronous communications between therapists and families [29,30,32,33,47,80] or technology-enhanced programs that augment face-to-face services conducted in the clinic [81]. Whilst synchronous TMH formats, which applies VC to deliver real-time treatment between therapists and families have been mainly applied to relatively small sample size of youth with anxiety-related conditions or OCD [70,71,82], depressed youth or externalizing youth [48]. Most of these TMH programs demonstrated a treatment response rates of 60–80% and a relatively comparability with clinic-based, face-to-face outcomes [30,82,83].

### 3.1. Anxiety Disorders

Using an internet-based approach, Spence et al. [34], developed and tested an online CBT-based program for youth anxiety disorders (BRAVE-ONLINE) in children (aged 7–12 years), comparing the internet-based approach with the clinical-based one, by demonstrating s significant reductions in anxiety symptomatology in both modalities. Another study by Spence et al. [30] carried out a randomized trial comparing computer-assisted CBT, in-person CBT and a waitlist control amongst 72 children affected with anxiety disorders (aged 7–14 years old), by delivering 6 sessions via the Internet and 6 in-person sessions in a clinic-based group treatment format. The findings of the study reported a significant reduction in anxiety symptomatology in both groups with active treatment, compared to the waitlist condition [30]. Accordingly, another Internet-based individual CBT program relative to a waitlist control in 73 children with anxiety disorders (aged 7–12 years) reported a significant reduction in anxiety symptomatology and a greater increase in adaptive functioning relative to the waitlist arm [33]. Khanna and Kendall [29] carried out a randomized trial of a computer-aided CBT protocol (Camp Cope-A-Lot[CCAL]), relative to in-person CBT and an Education/Support/Attention (ESA) control condition in 49 non-OCD anxious youth (aged 7–13 years old), by documenting a significant improvement in anxiety levels in both treatments compared to the ESA arm [29]. These improvements were maintained at 3-month follow-up. Similarly, the ‘Cool Teens’ program (i.e., a eight-session, CD-ROM-based program) demonstrated a clinically significant efficacy in improving anxiety symptomatology amongst adolescents in two studies [36,37]. A Sweden RCT using online CBT to reduce social anxiety, depression and general anxiety in high school students in a 9-week intervention consisting of psychoeducation, cognitive restructuring and exposure therapy, reported a significant decrease in social anxiety, social phobia, anxiety and depression levels which were maintained at the 1-year follow-up [37]. A Canadian RCT recruiting university students to test the effects of a self-help online intervention, including CBT, coaching, psychoeducation and relaxation together with audio files, pictures, videos and online activities, reported an improvement in depression, anxiety and stress levels, which were maintained at the 6-month follow-up [38]. An Australian 10-week online intervention consisting of five trail arms based on the presence of email or telephone reminders and consisting of 10 modules of CBT, psychoeducation, physical activity promotion, relaxation and mindfulness medication techniques, reported a significantly higher improvement in anxiety symptomatology at 12 months [39]. An Australian RCT recruiting students who were administered a 6-week online intervention at school consisting of CBT, psychoeducation, relaxation and physical activity named ‘Y-Worri/E-couch Anxiety and Worry Program’, described a significant improvement in anxiety levels after the TMH sessions [40]. A series of online attentional bias modification training RCTs were performed in the Netherlands, by recruiting high school students who reported a significant reduction in negative attentional bias for the visual search group [41,42,43]. A pilot study evaluating feasibility, acceptability and preliminary efficacy of real-time, Internet-delivered and family-based cognitive behavioural therapy (CBT) for anxious youth (*n* = 13) delivered to the home setting over the Internet using VC [44]. The intervention was feasible and acceptable to families who reported high treatment retention, high satisfaction, strong therapeutic alliance and low barriers to participation. The treatment showed efficacy with 76.9% of the intention-to-treat sample and 90.9% of treatment completers who achieved treatment response at the Clinical Global Impressions-Improvement Scale in post-treatment. These findings have been maintained at 3-month follow-up evaluation [44]. An online, randomized, non-inferiority design, synchronous VC to in-person counselling using solution-focused brief therapy in a sample of college students who were seeking help for mild-to-moderate anxiety, showed significant changes in Beck Anxiety Inventory scale and in social anxiety levels in both interventions without any significant differences in effectiveness of the two delivery methods [45]. A feasibility pilot study of a therapist-assisted online parenting strategies (TOPS) program addressed to parents whose adolescents (aged 12–17 years) are affected with anxiety and/or depressive disorders reported an overly acceptability and perceived usefulness of a web-based program which also involves parents/caregivers of affected adolescents [46].

Overall, a total of 4,038 patients were recruited in 18 studies, of which 3 studies providing findings and clinical observation during a short-term period (i.e., less than 1 month) [37,38,41], 8 studies followed up patients over a medium-term period (i.e., up to 3 months) [29,30,33,34,36,42,44], while 7 studies were carried out by collecting data during a long-term period (i.e., more than 3–6 months) [31,32,37,39,40,43,45]. Overall, six studies provided I-CBT treatment, one implemented an online-parenting CBT-based intervention, two studies administered a CD-ROM-based CBT online program, one study administered a self-help CBT-based online CBT plus psychoeducation, two studies administered I-CBT plus psychoeducation (of which one study which provided also a relaxing program and physical activity), one study administered a VS-based CBM-A, one a VS-based ABM training, one study administering a CBM-1 training, one study investigated the efficacy of a TMH-based FCBT program, one study administered SFBT, and one a TOPOS program. 

All studies demonstrated that TMH intervention determined an improvement in anxiety symptomatology, which seems to persist over the time. Moreover, literature so far published reported the overall superiority of the TMH-based interventions compared to placebo and, at least, a comparable efficacy to the face-to-face modality [29,30,31,32,33,34,36,37,38,39,40,41,42,43,44,45]. In addition, patients and their relatives reported from moderate to higher satisfaction rate following these TMH-based interventions.

### 3.2. Depression

TMH interventions, including both computer-based and therapist-delivered online psychotherapy (i.e., cognitive behaviour therapy, CBT), for depression in youth have been demonstrated to be efficacious, cost-effective and very comparable with face-to-face treatment [49,50,51,84,85,86]. Overall, despite the benefits of TMH interventions for depression, there is a lack of studies specifically focusing on adolescents and with longer-term follow-up, most of them displaying only short-term follow-up periods are represented by self-guided (i.e., delivered with an asynchronous modality) interventions [20]. Several controlled trials applied a self-directed Internet intervention for depression in a youth population (aka ‘MoodGYM’, i.e., delivered as a part of the high school curriculum) including five modules based on CBT focusing on prevention, education, and treatment addressed to depressive symptoms, by reporting an overall reduction of depressive symptomatology [47,52,53]. An Internet-based, Dutch, solution-focused, brief chat, prevention intervention (named ‘Grip op Je Dip’ or ‘Dutch for Master your Mood/MYM’) for adolescents with subclinical depression consisting in six 90-min online chat room sessions focused on CBT, BA and future planning, reported significant reduction in depression and anxiety which continued during the 6-month follow-up period [50]. PratenOnline, a one-on-one chat intervention with trained professionals running Solution-Focused Brief Therapy (SFBT) reported an improvement of depressive symptomatology which continued to decrease steadily over a 7.5-month follow-up [54]. The Project CATCH-IT (Competent Adulthood Transition with Cognitive-Behavioural and Interpersonal Training) is a primary care-centered interventional website designed to prevent depression in at-risk adolescents, by applying the principles based on CBT, Interpersonal Psychotherapy (IPT) and Behavioural Activation (BA) [55,56,57,58]. These studies reported a significant improvement in depressive symptomatology, loneliness scores and hopelessness scores which persisted from the 6-month to 2.5-years of follow-up [57,58]. A pilot study evaluated an online intervention for at-risk school students, by reporting a reduced suicidal ideation, hopelessness and depressive symptoms [59]. A RCT on a spirituality informed e-mental health tool as an intervention for major depressive disorder in adolescents and young adults and a RCT on a school-based CBT program described an improvement of depression and suicidality [60,61]. A single-group pilot study was carried out to evaluate a moderated online social therapy intervention named ‘Rebound’ for depression relapse prevention in young people, by reporting a significant improvement at Montgomery-*Å*sberg Depression Rating Scale (MADRS) (*p* = 0.014) [62]. An Australian intervention for youth called the DEAL Project evaluated co-occurring depression and alcohol misuse, by the application of a 4-week online intervention including four 1-h modules in the areas of CBT, MI, psychoeducation, BA, relaxing and mindfulness, coping strategies [63]. However, the study did not report any significant differences between intervention and control group over a 3- or 6-month period of follow-up [63]. ‘Grasp The Opportunity’ took place in Hong Kong and comprised the CBT, BA and resiliency themes from the original CATCH-IT website, by recruiting 257 Chinese youth who were asked to work through 10 online depression prevention modules and reported benefit at 12-months follow-up in the improvement of depressive symptomatology [64]. A randomized controlled trial compared a fantasy videogame named ‘SPARX’, applied to help adolescents with depressive symptoms over 7 weeks, to a control group, described a significant reduction in depressive symptomatology at the 12-month follow-up [65]. A RCT randomized a low-intensity CBT (LI-CBT) intervention versus self-help control arm in a sample of subclinical depression of university students, by reporting significant improvement in depression and anxiety at 2 months and over a 12-month period of follow-up [66]. An online 10-week active intervention, named ‘Image and Mood’ (aka ‘IaM’), consisting of CBT, IPT, BA, stress management and problem solving, was applied on female students at very high risk of developing an eating disorder with comorbid depression [67]. There was a significant improvement of eating and depressive symptomatology in iCBT group compared to the control group with a maintenance up to 1-year of follow-up [67]. A multicenter, randomized controlled E-motion trial, belonging to the German ProHEAD consortium (Promoting Help-seeking using E-technology for Adolescents), investigated the efficacy and cost-effectiveness of two online interventions to reduce depressive symptomatology in high-risk children and adolescents with subsyndromal symptoms of depression in comparison to an active control group [51]. The first intervention group complete the iFightDepression^®^ (iFD^®^ tool) which is an online, clinician-guided, self-management program aimed to help subjects with mild-to-moderate depression to self-manage their symptoms [68] whilst the second intervention group receive a clinician-guided, online, group chat intervention based on a CBT approach. Both groups are compared to a control intervention group who access to structured online psychoeducational modules on depression, however, the results of the study should be still published [51]. An observational 18-month TMH program by Fairchild et al. [9] reported an increased access to care for youngsters dealing with suicidality, depression, and anxiety in rural emergency departments. Similar findings have been previously reported in a study evaluating TMH resource usage by high school students, which reported that teens who reported depression symptoms, higher stress or suicidality were less likely to talk to a parent about stress or psychological distress but more likely to use TMH services or an online counselor to seek help [21]. A feasibility and acceptability study investigating a web-based model, including Skype, to screen and provide psychiatric consultation to 285 (out of 972 of the total sample) depressed college students, who reported the tool helpful in helping them understand their depression (76.4%) and thought that psychologists and psychiatrists could successfully see patients via VC (88.2%) [69]. 

In conclusion, a total of 8432 individuals were recruited from 24 studies here retrieved and analyzed on depression. Of which, 7 studies were conducted with a short-term follow-up (i.e., less than 1 month), 7 with a medium-term follow-up (i.e., up to 3 months) whilst 8 studies with a longer-term follow-up period. Among all studies, one study was based on a regional TMH work, one was based on a self-direct I-CBT program, one was based on W-CBT, one on VC-CBT, five studies were carried out to administer I-CBT intervention, two were based on a clinical-guided self-management program, one on a W-SFBT, five studies were based on a BA/MI intervention plus ICB, IPT and community resiliency concept, one study was performed to administer a I-CBT plus BA, MI, psychoeducation, relaxing, mindfulness, and coping strategies, one study was based on a spirituality-based e-mental health intervention, one study was based on online social networking, one study administered a videogame SPARX, one study on LI-CBT, one study on ICBT plus IPT, BA, stress management and problem solving, and one study was based on an online depressive toolkit. Overall, These THM-based interventions reported a good improvement in depressive symptomatology, which was maintained over the time, a superiority compared to placebo and a comparable efficacy to other types of face-to-face interventions. Moreover, these types of THM appear to be acceptable and feasible to patients and their relatives.

### 3.3. Obsessive-Compulsive Disorder (OCD)

VC interventions may be superior to other remote interventions (i.e., self-help, telephone-mediated psychotherapy or Telepsychiatry) as they may enhance face-to-face element of therapy and increase the accountability and adherence of patient [70,71,72,73,74,75,76]. However, most of studies carried out on OCD patients have small samples and intervention utilized expensive VC equipment [72,73]. A Swedish clinical trial named BiP (*BarnInternetProjektet*) OCD consisting in a 12-week, 12-chapter Internet-based intervention of CBT and psychoeducation delivered through film, exercises, animation and interactive scripts addressed to adolescents and their parents [75]. The intervention demonstrated an improvement in OCD symptomatology, which was maintained at the 6-month follow-up [75]. A RCT non-inferiority trial compared face-to-face CBT with telephone-CBT (TCBT) in a sample of 72 adolescents with OCD, by reporting a significant improvement in OCD and depressive symptomatology that persisted until 6-month follow-up [76]. An evidence-based treatment protocol for real-time delivery over webcam of CBT (W-CBT) intervention compared to a 4-week waitlist period reported a treatment response in OCD symptomatology in 81% of the youth sample with OCD with W-CBT compared to the waitlist arm with OCD improvements, which were maintained at a 3-month follow-up [70]. A pilot study evaluated an Internet-delivered family-based CBT intervention on a sample of early-onset OCD young people, by reporting an OCD symptomatology improvement and global severity improvements from pre- to -post-treatment [71]. An open label study explored the acceptability, feasibility and effectiveness of a newly developed enhanced CBT (eCBT) to children and adolescents [77]. The eCBT includes VC sessions, such as supervision and guided exposure exercises at home in addition to face-to-face sessions), an interconnected app designed for children, their parents and the therapists and an online assessment with direct feedback to patients and the therapist. The study was applied to 30 children (aged 7–17) with a primary diagnosis of OCD according to the DSM-5 criteria and their parents. The findings are still ongoing [77]. Overall, among 8 papers here retrieved, 2 were short-term, 4 medium-term, 1 with a long-term follow-up while only one is still ongoing not providing preliminary results but only a research protocol. A total of 200 patients were considered. One study investigating the use of W-CBT, one VTC-FB-CBT, one a manualized-CBT, one an intervention based on I-CBT only while one study combining I-CBT plus psychoeducational intervention, one study administering a VC-mediated-ERP intervention and one an eCBT. Therefore, a comparison of these studies is limiting being represented by not homogeneous interventions online delivered for OCD. However, all seven studies reporting preliminary findings showed a significant improvement in OCD symptomatology, which persisted over time [70,75,77], a superiority over placebo [70] and a comparable efficacy compared to face-to-face interventions [76], particularly among those studies in which the sample was randomly assigned to the intervention or WLC/face-to-face modality. 

## 4. Discussion

Nowadays, TMH approaches using VC to hold real-time, remote treatments with a live therapist have shown increasing support for a range of youth mental health problems, including anxiety disorders, depression, schizophrenia, substance abuse, posttraumatic stress disorders, neurodevelopmental disorders, oppositional defiant disorder, autism spectrum disorders, family conflicts and externalizing behaviours, without any specific contraindications to its use for any disorder or age range [17,19,31,70,71,82,87,88,89,90]. Moreover, further studies demonstrated how implementing a web-delivered approach as a supportive and complimentary tool to treatment as usual interventions may potentially improve the individual’ predisposition to be engaged to online tools for the prevention, assessment, monitoring and delivering of psychiatric and psychotherapy treatments. For example, Toscos et al. [91] demonstrated, through the use of an immediate-response technology (IRT), able to gather mental health care data and educate youth on TMH resources, that after interacting with IRT, 43% of youths expressed their willingness to use online self-help resources, 40% of them an online therapist, and 29% an anonymous online chat. These findings demonstrated a statistically significant preference of TMH services particularly amongst females (*p* < 0.01) and those who reported higher depression and anxiety scores (*p* < 0.001). 

The advances in the quality and availability of desktop VC technologies together with an increasingly large and sophisticated evidence-based studies in the field of TMH, including randomized controlled trials (RCTs) demonstrating the efficacy of TMH interventions in the treatment of several mental disorders, facilitated the implementation of TMH interventions amongst youngsters with mental disorders [8,22]. However, despite these encouraging premises, there are still few studies, which specifically addressed the feasibility and effectiveness of TMH interventions amongst youngsters in naturalistic settings. Moreover, there are still missing guidelines specifically addressing TMH practice for each mental health disorder in CAP (i.e., treating youth depression or anxiety or bipolar disorder, etc. with TMH). Overall, most of studies here retrieved demonstrated the superiority of TMH interventions over the placebo or a comparable efficacy of TMH intervention to face-to-face modality with regards to studies on youth anxiety [29,30,31,32,33,34,36,37,38,39,40,41,42,43,44,45] depression [9,21,38,46,48,49,50,51,52,53,54,55,56,57,58,59,60,61,62,63,64,65,66,67,68,69,83,84,85,86] or OCD [70,71,73,74,75,76,77,87]. Computer-assisted CBT approaches have been proposed as a complementary or alternative modes of treatment delivery aimed at increasing access to therapy for many mental health problems, including youth anxiety disorders [29,30,31,32,33,34,36,37,38,39,40,41,42,43,44,45], depression [47,50,51,52,53,55,56,57,58,60,61,63,64,66,67] and OCD [70,71,76,77,87]. Overall, computer-assisted CBT approach appear to be acceptable to youngsters and their parents and feasible for implementation by providers, as well as it seems to produce significantly greater reductions in clinician-rated anxiety and greater improvements in overall functioning compared to control group [29,30,31,32,33,34,36,37,38,39,40,41,42,43,44,45,83]. Some studies also reported a sustained improvement in anxiety symptomatology over a longer time [30,40]. Furthermore, parent-focused Internet-delivered CBT appear to be as well effective as an early intervention in treating preschool-age children with anxiety disorders in a modified version of the BRAVE-ONLINE program [31]. Studies including mixed samples (i.e., individuals with anxiety and depressive symptomatology) similarly reported improvements in both clinical dimensions [37,40,41,42,43,46,83]. Moreover, both computer-based and therapist-delivered online psychotherapy (i.e., cognitive behaviour therapy, CBT) for youth depression have been demonstrated to be effective, cost-effective and very comparable with face-to-face treatment [49,50,51,84,85,86]. Overall, despite the benefits of TMH interventions for youth depression, there is a lack of studies specifically focusing on adolescents and with longer-term follow-up, with most of them which display only short-term follow-up periods which are self-guided (i.e., delivered with an asynchronous modality) [20]. Moreover, some studies also observed a sustained improvement in depressive symptomatology over the time [9,50,54,57,58,67]. In addition, a significant improvement in OCD symptomatology has been observed as well [70,71,75,76], also maintained over the time [70,76]. Despite these encouraging findings, there are several limitations, which should be here considered. Firstly, most of the studies here retrieved own extreme heterogeneous methodologies and study designs, including interventions delivered via TMH which are not always comparable (i.e., CBT vs IPT vs family-based interventions), with a different length of treatment (with some of them which do not provide a longer follow-up), without homogenous characteristics of the sample (i.e., some studies included only children, some included children and adolescents whilst others included only adolescents). Secondly, not all studies compared internet-delivered intervention vs face-to-face intervention vs placebo group. Thirdly, few studies own a large sample size, mainly being carried out on small sample sizes, with mixed samples (i.e., anxious and depressive individuals, some including both parents/caregivers and patients). Finally, some studies are only pilot study or evaluated the feasibility and acceptability of computer-assisted or internet-based CBT or TMH interventions, without reporting completed findings [29,46,69,77].Therefore, further studies should include longer term follow-up (in order to assess the effectiveness in naturalistic settings); larger sample sizes to examine mediators and moderators of outcomes following TMH interventions in youngsters affected with anxiety disorders, major depressive disorder or OCD; provide complementary parent self-report measures (in order to compare and integrate findings coming from self-report assessments provided by young people) and clinician-based assessments during each TMH session. Moreover, further studies should investigate and define specific standardized, age- and young-tailored Internet-delivered protocols address to youth anxiety disorders (i.e., social anxiety, generalized anxiety disorder, separation anxiety disorder, phobias, etc.), depressive disorders and OCD. 

Furthermore, despite the current increased use of telepsychiatry and telepsychotherapy due to the current COVID-19 pandemic, there is still limited knowledge and a missing formal clinician training and universally recognized certification in the field of TMH adolescent psychiatry [8]. Following the recommendation by Hilty et al. [87] for further clinical guidance on TMH particularly amongst the youngsters, the American Academy of Child and Adolescent Psychiatry (AACAP) proposed a practice parameter for Telepsychiatry with children and adolescents [88]. The AACAP in partnership with the American Psychiatric Association (APA) developed an online Child and Adolescent Telepsychiatry Toolkit in 2019 to address issues and concerns regarding the practice of Telepsychiatry with children and adolescents [89]. The toolkit, developed as complementary to the APA’s Telepsychiatry Toolkit for adults, includes a series of video series covering general topics in telepsychiatry (i.e., history, theoretical background, reimbursement and regulatory issues) and sections on training, practice and clinical issues, particularly focusing on CAP [89]. In 2017, the American Telepsychiatry Association (ATA) provided a clinical guidance for the delivery of child and adolescent mental health and behavioural services by a licensed health care provider through real-time VC [7,25]. Furthermore, in 2018, ATA and APA released a guide to assist mental health professionals in providing effective and safe mental health care regarding VC-based TMH [88,90,91]. 

In general, mental health professionals working with adolescents should be trained and acquire a set of competencies and skills essential for effectively practicing TMH interventions in CAP (Table 3) as well as overly trained in social media, mHealth, wearable sensors and asynchronous modalities [10,12,13,88]. Before starting a TMH session, clinicians should be able to introduce and clearly explain TMH modality to young patients and their parents, including what to expect and a basic explanation of the process, confidentiality and the needed equipment (Table 3) [6,22,54,88,89,90,91].

Overall, TMH services may greatly address access issues in adolescent mental health, by limiting unnecessary travel, reducing school absences and parental time off from work, bring specialty care to local communities, and improving outcomes by reducing delays in diagnosis and treatment [23]. This is particular relevant, whether the clinician considers that the current digitally native youngsters appear to be more comfortable in seeing the doctor by video and in receiving mental health services by online, as they are accustomed to being connected through the use of electronics, such as social networks, videogames, and interactive VC applications on mobile devices [92,93]. However, the appropriateness of a TMH intervention in Youth Mental Health should be individualized and tailored depending on the developmental stages, patient’s comfortability with the digital tool, parents’ preferences, and the availability of technical supports at the patient site as well as the telepsychiatrists’ resourcefulness and competences in delivering TMH interventions in Youth Mental Health [83,94,95,96,97,98,99,100]. Moreover, as there are no uniform shared regulations regarding TMH interventions, it should be a recommended clinical practice to consult own country’s laws and medical board guidelines and regulations, before initiating a TMH session in Youth Mental Health, particularly regarding the practice of prescribing by means of the Internet (i.e., e-prescribing), the informed consent and the privacy issues. Ideally, the prescriber psychiatrist should conduct at least one face-to-face psychiatric evaluation of the patient before prescribing a psychopharmacological treatment through TMH. Moreover, TMH in Youth Mental Health may largely vary by country’s jurisdiction and needs a proper dedicated protocol, particularly amongst adolescents in at-risk situations and in the treatment provided in foster care and correctional settings [101,102] (Table 3). 

Furthermore, TMH services should ensure privacy and data security. In fact, when a clinician decides to offer a TMH session, he/she should understand not only the labeling of encryption, but the publically available information about the encryption process and who could potentially access this information. Whether there are potential safety and privacy risks, the therapist should be encouraged to be transparent with the patient about limitations and pitfalls of technology used. Moreover, TMH protocols most often address the secure transfer of patient written information by fax and/or secure e-mails. Protocols should detail procedures for shared information between institutions for both paper and electronic health records associated with TMH care [96,101,102] (Table 4). 

More in details, psychiatrists should be aware about the need to firstly obtain a specifically designed informed consent for TMH services, e.g., it would ideally better whether the informed consent is distinct and separate from that referring to face-to-face visit. Moreover, families and the child/adolescent should be informed, during the process of obtaining informed consent, about the steps needed and the practice of TMH services, its benefits, and any risks that might be involved at the patient’s site, including occurring privacy and safety issues. In addition, ethical issues should be balanced and communicated to the parents and young patient before commencing TMH services and keep sure that all (including parents/caregiver(s)) give their consents to proceed with TMH services [88,89,90,91,96,102] (Table 4).

Despite the lack of a complete clinical guidance for TMH for each youth mental disorder, there is currently available good telepsychiatry toolkits [89] and the ATA Child and Adolescent TMH Guideline [92,93] which provide a practical guide for apps, asynchronous, e-mail, e-consultation, monitoring, social media, text and wearables useful in the field of young mental health [25]. Furthermore, most psychiatrists and mental health professionals may be resistant in integrating TMH into their practice mainly due to the lack of relevant education, training, clinical experience and exposure to the technology during their training program. Moreover, specific training and education programs in TMH appear to be country-specific and there is a lack of a dedicated program of TMH in Youth Mental Health. Beyond these resistances and difficulties encountering by mental health professionals working with adolescents, another key component of TMH practice should include the need to ensure integrity (i.e., an accurate, honest and truthful clinical and scientific practice of mental health care) during a TMH session which may be missing in some not enough educated contexts [88,89,90,91,96,102].

## 5. Conclusions

Overall, VC-based or audio call TMH interventions appear to be feasible, preferred and easy to apply for youth mental health in the treatment and monitoring of youth depression, anxiety and OCD. However, it is still needed to shape practice models and tailored modalities, to ensure that the quality of care meets the standards of traditional face-to-face care and guarantees the safety and protection of adolescents and their parents. However, there is the still a need to develop national and regional TMH Resource Centers able to aid, provide training, education and information to organizations and individuals, both in academic, public and private practice settings, who are interested or confident in providing TMH care. Furthermore, TMH programs should be integrated and adequately trained within psychiatric training programs, by deepening multifaceted aspects of Youth Mental Health, including how to provide school-based TMH, TMH in juvenile correctional settings and in daycare centers. Finally, mobile health, sensors, social media, and other TMH services should be ideally integrated into clinical workflow for youth mental health. Further research directions, particularly RCTs, should be specifically addressed to youth mental health and the application of TMH by considering different levels of functioning and maturation of young people, as well as their diagnosis. 

## Figures and Tables

**Table 1:** Psychiatric services delivered by TeleMental Health.

Psychiatric Evaluation	Neurobehavioral status examination Psychiatric diagnostic service
Psychiatric Crisis service	Psychological first aid Psychiatric first aid Suicidal risk assessment
Initial Outpatient Visit	Outpatient new patient visit
Established Outpatient Visit	Subsequent hospital care Prolonged service inpatient Prolonged service outpatient visit
Teletherapy	Any nonpharmacological, psychotherapeutic interventions delivered through videoconferencing, including psychotherapy, social skills training, mindfulness training, activation behaviour, etc.

**Table 2:** Summary of retrieved papers.

Study	Sample Features	Intervention	Advantages	Findings
Anxiety				
[29]	49 children (M 33; F 16), aged 7–13 yy, with a principal anxiety disorder	The sample was randomly assigned to CCCAL, a computer-assisted CBT for anxiety in youth, to individual CBT, or to a computer-assisted education support and attention (CESA) condition. Independent diagnostic interviews and self-report measures were completed at pre-, post-treatment, and 3-month follow-up.	Findings support the feasibility, acceptability and beneficial effects of CCAL for anxious youth.	At post-treatment ICBT or CCAL children showed significantly better gains than CESA children. 70%, 81% and 19% respectively, no longer met criteria for their principal anxiety diagnosis. Gains were maintained at follow-up, with no significant differences between ICBT and CCAL. CCAL and ICBT has higher satisfaction than CESA.
[30]	115 clinically anxious adolescent, aged 12 to 18 yy (M 47; F 68) and their parents	The sample was randomly assigned to an online CBT therapy for youth with anxiety disorders (BRAVE-ONLINE) versus clinic delivery CBT or WLC. The BRAVE-ONLIne program is delivered through 10 weekly sessions for adolescents and five sessions for parents, each of approximately 60 min in duration. At 1 month and 3 month following treatment, booster sessions are provided for parents and adolescents to consolidate previously acquired skills. Adolescents also receive 15-min telephone calls following sessions 5. Prior to treatment, each family was assigned to a therapist who monitored their progress. The outcomes were evaluated through ADISC/P, CGAD, SCAS and a 5-item questionnaire to evaluate the treatment expectancy and satisfaction.	Online CBT, with minimal therapist contact, for adolescent anxiety disorders offer an efficacious alternative to clinic-based treatment.	Assessment at 12 weeks post-baseline showed significantly greater reductions on anxiety diagnosis and anxiety symptoms for both online e clinic CBT compared to waitlist control. 68% of adolescents in the online CBT group no longer met criteria for the principal anxiety diagnosis at 12-month follow-up compared with 80.6% in the clinic group. Satisfaction ratings by adolescents were equivalent for online-CBT and clinic-CBT, whereas parents indicated slightly higher satisfaction ratings for the clinical format.
[31]	52 pre-school r children (M 24; F 28), aged 3–6 yy, with clinical anxiety disorders (ADIS-C)	The sample was randomly allocated into an internet-based, parent-focused CBT (NET) versus WLC The intervention group consisted of 6 parent online sessions and 2 boosters BRAVO-ONLINE for children, together with phone and video consultation and weekly email	Intervening so early in the development of anxiety has the potential to prevent problematic trajectories that frequently results in life-long suffering. The use of internet programs has the potential to reach many more families than face-to-face therapy.	At post-treatment 39.1% of the NET children compared to 25.9% of the WLC were free of their primary diagnosis. By 6-month follow-up for the complete sample, 70.6% of children were free of their primary diagnosis. Satisfaction levels, as measured by a 8-item questionnaire, reported moderate to high rate satisfaction
[32]	433 parents of children aged 3 to 6 yy, with an inhibited temperament (OAPA)	The sample was randomly assigned to the online parenting program or to a 24-week WLC. The online program consists of 8 interactive modules providing strategies that parents can implement and a telephone consultation support with psychologists.	Offering an online adaptation of the Cool Little Kid parenting program appears to be effective in reducing anxiety in inhibited young children. The follow-up at 12 and 24 months guaranteed to collect longitudinal data.	The intervention group showed significantly greater improvement over time in child anxiety symptoms compared to the control group (*d* = 0.38), greater reductions in anxiety life interference (*ds* = 0.33–0.35) and lower rates of anxiety disorders than the control group (40% versus 54%).
[33]	73 children (33 M; 40 F) with anxiety disorders (ADIS-C/P), aged 7–12 yy, and their parents.	The sample was randomly assigned to NET or WLC. The NET consists of the BRAVO-ONLINE. Satisfaction with the program was measured, after 10-week, through an 8-items rating scale. Two booster sessions were conducted 1 and 3 month following the end of treatment.	Approaches incorporating only minimal therapist contact for CBT treatment of child anxiety disorders can produce clinically and statistically significant reductions in anxiety. This type of approach is well accepted by families as shown from the satisfaction questionnaire.	Children in NET intervention showed small but significantly greater reductions in anxiety symptoms and increases in functioning than WLC participants. These improvements were enhanced during the 6-month follow-up period, with 75% of NET children free of their primary diagnosis.
[34]	72 clinically anxious children (M 42; F 30), aged 7–14 yy	The sample was randomly allocated to an online CBT based program, to clinic-based on or to WLC. The online programme consists of 10 child sessions and 6 parent sessions, plus booster sessions at 1 and 3-months post-treatment. The child group sessions were 60 min in duration, conducted once a week for 10 consecutive weeks. Parent sessions were also 60 min in duration, conducted in a group format over 6 weeks.	The internet treatment content was highly acceptable to families, with minimal dropout and a high level of therapy compliance.	Children in the clinic and clinic-plus-internet conditions showed significantly greater reduction in anxiety from pre-to post-treatment compared to WLC. Improvements were maintained at 12-month follow-up for both therapy conditions.
[35]	5 adolescents (M 1; F 4), aged 14–16 yy, with anxiety disorder (ADIS-C)	Participants used the 8-module Cool Teen CD-ROM over a 12-week period on their home computer. Every 2 weeks, they received a brief telephone call from a clinical psychologist.	Participants were generally satisfied with multimedia content, the modules and the delivery format of the program.	Two participants no longer met diagnostic criteria (ADIS) for at least one clinical anxiety disorder. These participants no longer met diagnostic criteria for any clinical anxiety disorder at 3-month follow-up.
[36]	43 adolescents (M 16; F 27), aged 17–17 yy, with primary diagnosis of anxiety (ADIS-C/P & SCAS-C/P)	Adolescents were randomly allocated to the Cool Teens program, an eight-session, CD-ROM-based program for anxiety management, or a 12-week WLC. The program uses a combination of multimedia formats (text, audio, illustrations, cartoons, video) and, also telephone sessions.	The Cool Teens program is an efficacious option for the treatment of adolescent anxiety.	Adolescents in the Cool Teens condition, compared with those on the WLC, were found to have significant reductions in the total number of anxiety disorders. There was also a significant reduction in the clinical severity of the primary anxiety disorder in the Cool Teens group compared with the WLC group. At post-treatment, 41% of participants in the Cool Teens group no longer met diagnostic criteria for their primary anxiety disorder. This reduction was also maintained at the 3-month follow-up assessment.
[37]	19 high school students, aged 15–21 yy, with social anxiety disorder (SPSQ-C & MADRS-S)	The sample was randomly allocated into NET or WLC. The NET consists of 9 weeks of an Internet-delivered CBT.	Internet-delivered CBT could be an option to treat high school students although strategies to increase compliance should be found.	Significant improvements were found on measures of social anxiety, general anxiety, and depression. The average number of complete modules in the CBT program was low.
[38]	66 distressed university students (DASS-21)	The students were randomly assigned to either Immediate Access or a 6-week Delayed Access condition. The online program consists of 5 core modules. Each module is organized in a multimedia workbook format (including psycho-education, real-life examples, videos, audio files, pictures, phone call, weekly email). A 10-items questionnaire at the end of each module.	An individual-adaptable, internet-based, self-help programs can reduce psychological distress in university students.	Access participants reported significantly greater reductions in depression, anxiety and stress, in comparison to participants waiting to do the program. These improvements were maintained at six-month follow-up.
[39]	558 internet users, recruited via the Australian Electoral Roll	The sample was randomly assigned to 5 arms: Group 1: received combined psychoeducation, ICBT, physical activity and relaxation Group 2: received the identical Web program plus weekly telephone reminder calls Group 3: instead telephone reminder, weekly email reminder Group 4: received a placebo website. Group 5: placebo website plus telephone calls.	This trial is not able to demonstrate the preventative effects of the website on anxiety symptoms as measured by the GAD-7.	GAD-7 symptoms reduced over post-test, 6-month, and a 12-months follow-up. There were no significant differences between Group 4 and Group 1,2,3, or 5 at any follow-up.
[40]	A three-arm cluster stratified randomised controlled trial take in consideration 1767 students (M 37.2%; F 62.8%) about anxiety disorder	Each student was randomly assigned to receive externally-supported intervention, teacher-supported intervention or WLC. The online program is based on the e-couch Anxiety and Worry program, an online program for generalised anxiety, delivered over 6 sessions and consists of two core sections psychoeducation and CBT, relaxation and physical activity. Outcomes were measured by the GAD 7-item scale, MINI, CES-D, ASI, PSWQ.	The e-couch Anxiety and Worry program did not have a significant positive effect on participants.	At post-interventions, 6 and 12-month follow-up no significant differences were observed between the intervention and control conditions for generalized anxiety (*d*= 0.14 to 0.15), social anxiety (*d=* 0.04−0.23), anxiety sensitivity (*d* = −0.07 to 0.07), depressive symptoms (*d =* −0.05 to 0.04) or mental wellbeing (*d* = −0.06 to −0.30).
[41]	340 adolescents (M 42.4%; F 57.6%), aged 11–18 yy, recruited from 14 regular high schools in the Netherlands	To evaluate the efficacy of CBM-A, the sample was randomly allocated to eight sessions of a dot-probe (DP), or a visual search-based (VS) attentional training, or one of two corresponding placebo control conditions and received 8 sessions of an online training over four weeks.	More research is needed to investigate and improve the efficacy of CBM-A in adolescents.	One year of follow-up, showed that VS training was effective in reducing attentional bias, compared to DP training. Primary and secondary emotional outcome measures revealed a general improvement in emotional functioning irrespective of condition. CBM-A in its current form, seems to be ineffective in reducing anxiety or depressive symptoms in unselected adolescents.
[42]	108 adolescents (M 33.3%; F 66.7%), aged 11–19 yy, with symptoms of anxiety and/or depression (SCARED & CDI)	The current study investigated the effects of eight online sessions of visual search (VS) ABM, following four weeks, compared to both a VS placebo-training and a no-training control group online training sessions.	There is no evidence for the efficacy of online visual search ABM in reducing anxiety or depression or increasing emotional resilience in selected adolescents.	VS-ABM training reduced attentional bias compared to both control groups, with stronger effects for participants who completed more training sessions.
[43]	173 participants	Adolescents were randomized over one of two training groups: a CBM-I training, consisting of eight online sessions, or a placebo-control training. Emotional measures were administered both pre- and post- training, and at three, six and 12-months follow-up	Results suggest that interpretation training as implemented in this study has no added value in reducing symptoms or enhancing resilience in unselected adolescents.	Compared to placebo, interpretation training marginally increased positive interpretations. Change in interpretation bias, baseline interpretation bias, stressful life events, or number of training sessions completed did not moderate the effects on anxiety or depression.
[44]	13 youth (M 6; F 7), aged 8–13 yy, with a primary/co-primary anxiety disorder diagnosis and their mothers	Participants were randomly assigned to 1 of 3 baseline intervals (two-week baseline interval, three-week, four-week). The online intervention consists of 16 weekly sessions of TMH-FCBT. 44-item parent-report (BTPS) and a 36-item parent-report (WAI) were administered at week 4, mid treatment, week 12 and post-treatment, to evaluate feasibility and acceptability. An 8-item (CSQ-8) questionnaire was administered to evaluate satisfaction. The outcomes were evaluated through ADIS-C/P; CGI; MASC-C/P; CBCL; FACLIS.	Videoconferencing treatment formats may serve to improve the quality care of youth anxiety disorders.	○ The intervention was feasible and acceptable to families who reported high treatment retention, high satisfaction, strong therapeutic alliance and low barriers to participation. ○ The treatment showed the efficacy with 76.9% of the intention-to-treat sample and 90.9% of treatment completers. ○ Gains were largely maintained at 3-month follow-up.
[45]	49 undergraduate students (M 4; F 45) who were seeking counseling for mild to moderate anxiety	Undergraduate participants were randomly assigned to online, synchronous video counseling or in-person treatment for anxiety using solution-focused brief therapy (SFBT). The outcomes were evaluated with BAI and CCAPS.	The findings provide support for the treatment of college students with anxiety with SFBT through online, synchronous video counseling.	○ In both types of interventions have been found changes in BAI and in social anxiety levels, even though without any significant differences in the effectiveness of two delivery methods (*p* = 0.580).
[46]	Develop a Therapist-assisted Online Parenting Strategies (TOPS) program that is acceptable to parents whose adolescents have anxiety and/or depressive disorders, using a consumer consultation approach	TOPS intervention was developed via three linked studies. ○ Study 1: involved content analysis of feedback from participants (*n* = 56) who received a web-based preventive parenting intervention called Partners in Parenting (PiP), as part of a randomised controlled trial. ○ Study 2: involved stakeholder consultations with parents of adolescents aged 12–17 yy (*n* = 6) and mental health professionals (*n* = 28), to identify adaptations to PiP. ○ Study 3: a pilot of the resulting TOPS program with professionals (*n* = 10) and a small sample of parents (*n* = 3) to assess the acceptability of the program content and format.	This study provided preliminary support for the feasibility, acceptability and perceived usefulness of the TOPS program	○ Study 1 indicated a need for an enhanced program for parents whose adolescents are experiencing anxiety and depressive disorders, while findings from Study 2 informed the content of the new TOPS program. ○ In Study 3, mental health professionals endorsed the structure and content, while parents affirmed the acceptability of the TOPS program. ○ Feedback from Studies 2 and 3 indicated that the therapist-coach was a valuable resource to (i) provide parents with strategies that are associated with the alleviation of adolescent anxiety and depression, (ii) discuss difficulties in implementing these strategies, (iii) assist parents with overcoming these difficulties; and (iv) support the development of a relapse prevention plan. ○ Professionals felt that the TOPS program would broaden parental knowledge about how to recognise and respond to symptoms of clinical anxiety and depression in their adolescent.
**Depression**				
[9]	Observational an 18-month program for children less than 18 years (n = 87) who received physical and mental health assessment by ED physician	Wabash Valley Rural Telehealth Network utilizes an on-demand design with a centralized “hub” of medical providers that delivers specialty based psychiatric care via a regional telehealth network.	Decreasing waiting time in ED for those children and adolescents who need a CAP specialist in remote areas without CAP.	○ 49% of children presented with depression or anxiety-related disorders; 46% with suicidal ideation/attempt or self-harm; 5% with substance abuse/overdose. ○ 63% of cases treated are in the 14–17 age range, mainly females (66%) and white (98.5%).
[47]	1477 students (M 651; F 826), aged 12–17 yy, from 32 schools across Australia	Each school was randomly allocated to the online, self-directed CBT program (MoodGYM) or WLC. The MoodGYM program consists of 5 modules, one module each week. Classroom teacher supervised the students. The outcome was measured with RCMAS and CES-D.	Although small to moderate, the effects obtained in the current study provide support for the utility if prevention programs in schools.	○ At post-intervention and 6-month follow-up, participants had significantly lower levels of anxiety than the WLC group (*d* = 0.15–0.25). ○ The effects of the MoodGYM program on depressive symptoms were less strong (*d* = 0.27–0.43).
[48]	38 families e 28 children (20 M; 6 F), aged 8–14 yy, with childhood depression (K-SADS-P & CDI)	The patients were randomly assigned to a face-to-face CBT versus VC-CBT. The current project was an adjunct to Kansas University Medical Center’s TeleKidcare project and consists of 8 weekly sessions.	NA	○ The CBT treatment across both delivery methods was effective in decreasing depression. 82% of the 28 children no longer met the depression criteria at the end of the study. ○ The VC group reported a greater decrease in depressive symptoms over time as compared do the F2F group. ○ Good satisfaction with both types of treatment was found through the Telemedicine Satisfaction Questionnaire.
[49]	297 patients (M 32%; F 68%), aged 18–75 yy, having a new episode of depression (BDI & CIS-R)	The study evaluated the cost-effectiveness of therapist-delivered internet CBT compared with usual care for primary care patients with depression through randomised controlled trials. The outcomes were measured through BDI and QALYs.	This type of therapy appeals in particular to those who like to write their feelings down, those who value the opportunity to review and reflect on the dialogue of the therapy session, and those who prefer the anonymity offered by this method of delivering CBT.It could be an alternative to face-to-face treatment for those whose first language is not English.The intervention may also be useful when traveling is difficult or expensive because of rurality, disability or social phobia.	○ Online CBT was more expensive than usual care, although the outcomes for the CBT group was better.
[50]	244 young people, aged 16–25 yy, with depressive symptoms (CES-D)	Individuals were randomly assigned to a Web-based group course called Grip op Je Dio (Master Your Mood), designed for people aged 16 to 25 yy with depressive symptoms, and to a WLC. The course is based on six online-chat sessions. Outcomes were measured with CES-D; HADS.	MYM course was effective in reducing depressive and anxiety symptoms and increasing mastery in young people.	○ The MYM group showed significantly greater improvement in depressive symptoms at 3 months than control group (*p* < 0.001). ○ The MYM group also showed greater improvement in anxiety (*p* < 0.001) and mastery. ○ Improvements in the MYM group were maintained at 6 months.
[51]	363 children and adolescents, aged ≥ 12 yy, with subsyndromal symptoms of depression (PHQ-A) recruited at five sites across Germany, by the German ProHEAD consortium.	The sample was randomly allocated to a clinical-guided self-management program (iFightDepression), to a clinical-guided group chat intervention based on CBT approach and to a control intervention (psycho-educational website). All interventions had a duration of six weeks. The first intervention consists of six core modules. Each module comprises written information, worksheets, exercise, psychological training. The second intervention consists of six 90-min group chat sessions, once a week.		○ The results of the study should be still published.
[52]	79 boys, aged 15–16 yy	The boys were allocated to either undertake a CBT therapy Internet program (MoodGYM) or to standard personal development activities. The MoodGYM program was based on 5 modules that took 30–60 min, one module per week, and a tutor group meet weekly. Outcomes were measured before commencement, post-program and 16-weeks post-program with CES-D, CASQ-R, RSES.	Considering the high drop-out rate there is the need to review the appropriateness and difficulty of the material as well as the formats used in Internet programs.	○ There were no significant between-group differences in change scores pre- to post or pre-to follow-up. ○ Both groups showed improvements in their beliefs about depression at follow-up, with the control group showing moderate relative benefits. ○ The risk of being classified as vulnerable to depression reduced by 17% in the MoodGYM group at post-treatment compared with non-change risk for the control group. These reductions in risk were not sustained at follow-up.
[53]	157 girls, aged 15–16 yy, come from a single sex school in Canberra, Australia	Students were allocated to undertake either MoodGYM or their usual curriculum.	MoodGYM brings benefit on self-reported depressive symptoms, even though it had low rates of completion. Overall, findings offered encouragement to the development of effective school-based interventions for reducing depression in adolescents via the Internet.	○ MoodGYM produced a significantly faster rate of decline in depressive symptoms over the trial period than the control condition. ○ The effect size for MoodGYM was not significant immediately after the intervention (*d* = 0.19) but was moderate and significant 20 weeks after (*d* = 0.46). ○ Girls with high depression scores before intervention showed the strongest benefits on self-reported depression at follow-up. ○ There were no significant intervention effects on depression status, attributional style, depression literacy and attitudes.
[54]	263 young individuals aged 12–22 yy with depressive symptoms (CES-D)	Young individuals were randomly assigned to a web-based SFBT intervention (PratenOnline) or WLC. Outcomes were evaluated by CES-D after 9 weeks and 4.5 months.	Chat condition demonstrated a reliable and clinically significant improvement at 4.5 months, but not yet at 9 weeks.	○ The intervention group showed significantly improvement compared to WLC in depressive symptoms at 9 weeks and 4–5 months on the CES-D (*d* = 0.18 and 0.79, respectively). ○ A reliable and clinically significant change in depression was significantly larger for the SFBT intervention at 4.5 months only. ○ At 7.5 months, SFBT group shower further improvements.
[55]	84 adolescents, aged 14–21 yy, at risk for developing major depression (PHQ-A) were selected through the CATCH-IT project	The sample was randomly allocated to two groups: brief advice (BA; 1–2 min) + Internet program versus motivational interview (MI; 5–15 min.) + Interne program. In the MI group, the physician used a non-directive approach to help the adolescent develop a favorable cost/benefit assessment toward completing the intervention and building resiliency. In the MI group also received three motivational phone calls from the case manager. The internet intervention included 14 Internet-based modules based on CBT, IPT and a community resiliency concept. A workbook was given to the parents of adolescents under age 18. Outcomes were evaluated with a questionnaire created by the authors, CES-D and ANDHEALTH questionnaire.	In the BA condition, the physician takes a directive approach and advises the adolescent that he is experiencing a depressed mood and refers the adolescent to the CATCH-IT internet site.	○ Both groups demonstrated declines in depressive mood. ○ Both groups demonstrated increases in social support by peers and reductions in depression related impairment in school.
[56]	84 adolescents, aged 14–24 yy, recruited when they visited the primary care provider for risk of depressive disorder, as well as through advertisements posted in and around the clinics.	The sample was randomly assigned to primary care physician motivational interview (MI) + Internet program versus brief advice (BA) + Internet program. In the BA condition the physician takes a directive approach and advises the adolescent that he is experiencing a depressed mood and refers the adolescent to the CATCH-IT internet site. In the MI group, the physician used a non-directive approach to help the adolescent develop a favourable cost/benefit assessment toward completing the intervention and building resiliency. In the MI group also received three motivational phone calls from the case manager. The Internet intervention consists of 14 modules based on BAC, CBT, IPT and a community resiliency concept model. Outcomes were measured with PHQ-A and CES-D.	This tool may help extend the services at the disposal of a primary care provider and can provide a bridge for adolescents at risk for depression.	○ Both groups substantially engaged the Internet site. ○ For both groups CES-D scores declined from baseline to 12 weeks (MI, 52% to 12%; BA 50% to 15%). ○ The MI group demonstrated declines in self-harm thoughts and hopelessness and was significantly less likely than the BA group to experience a depressive episode (*p* = 0.044) or to report hopelessness (*p* = 0.044) by 12 weeks.
[57]	84 participants (M 43.4%, F 56.6%), with mean age of 17.47 yy, were recruited by screening for risk of depression in 13 primary care practices	Randomized comparison of two approaches in engaging adolescents with the Internet intervention: prime care physician (PCP) motivational interview (MI) + CATCH-IT Internet program versus PCP brief advice (BA) + CATCH-IT Internet program. Outcomes were measured with CESD-10, PHQ-A.	It would be useful to make these interventions more accessible to adolescents given their good effectiveness.	○ Depressive symptoms scores declined from baseline to 6 weeks with these statistically significant reductions sustained at the 6-months follow-up in both groups. ○ By 6-months, the MI group demonstrated significantly fewer depressive episodes and reported less hopelessness as compared to the BA group.
[58]	83 adolescents recruited from 12 primary care sites across Southern and Midwestern United States	Adolescents recruited were randomly assigned to a version of the CATCH-IT intervention: primary care provider (PCP) motivational interview (MI) + Internet program or PCP brief advice (BA) + Internet program. Outcomes were measured with CESD-10, PHQ-A.	The tool may help extend the services at the disposal of a primary care provider and can provide a bridge for adolescents at risk for depression.	○ Both groups demonstrated significant decrease in depressed mood, loneliness, and self-harm ideation. ○ Fewer participants in the MI group, after one-year, had experienced a depressive episode.
[59]	34 students were recruited from nine schools	A pilot study employed a pre-test/post-test design with 8-week intervention based on the Reframe Internet-based program interventions. It consists of 8 modules, based on CBT, each of which takes around 10–20 min to complete.	The finding are promising and suggest that young people at risk of suicide can safely be included in trials as long as adequate safety procedures are in place.	○ 21 young people completed the intervention and they found a reduced suicidal ideation, hopelessness and depressive symptoms. ○ Participants reported enjoying the programme and said they would recommend it to a friend.
[60]	62 participants with major depressive disorder were defined by two age subgroups: adolescents (*n* = 31), aged 13–18 yy (CDRS-R), and young adults (*n* = 32), aged 19–24 yy (HAMD).	Participants in each subgroup were randomized into the intervention group or WLC. The intervention consists of an 8-week online spirituality informed e-mental health intervention (LEAP project) in which there is the use of fresh graphic designs, video clips, music clips, youth autobiographical story, off-line activity, relaxation techniques, online journals and others.	Spirituality is increasing as an important consideration in mental Health and mental health interventions.	○ At baseline, there was no statistical difference between study and WLC for both age subgroups. After the intervention, depression severity was significantly reduced. ○ Self-concept improved significantly for younger participants immediately after the intervention and over time in the study arm. In older participants, change was minimal.
[61]	3224 youth (M 1676; F 1568), aged 11–18 yy, selected from 5 schools in the Red Deer Public School system	All these students were entered in the EMPATHY program. They were screened for depression, suicidality, anxiety, use of drugs, alcohol, or tobacco, quality of life and self-esteem. Additionally, all students in Grades 7 and 8, also received an 8-session CBT based program. The intervention consisted of an interview with the student and their family followed by offering a guided internet-based CBT program. Outcomes were evaluated using PHQ-A, HADS, RSE, K-10, CRAAFT questions.	Suggesting that a multimodal school-based program may provide an effective and pragmatic approach to help reduce youth depression and suicidality.	○ The 2790 students who completed scale at both baseline and 12-week follow-up showed significant decreases in depression and suicidality. ○ There was a marked decrease in the number of students who were actively suicidal (from 125 to 30). ○ Of the 503 students offered the CBT program, 163 (32%) took part and this group had significantly lower depression scores compared to those who did not.
[62]	42 youth (M 22; F22), aged 15–25 yy, affected by depression in partial or full remission	Participants had access to the Rebound platform for at least 12 weeks, including the social networking, peer and clinical moderator and therapy components. Rebound is an online platform, based on the MOST model and included: peer-to-peer online social networking, individually tailored interactive psychosocial interventions and involvement of expert mental health and peer moderator.	These types of online social networking are well appreciated by the young people, and further studies would be needed to perfect their development.	○ There was High system usage. Almost 70% of users had >10 logins over the 12 weeks with 78.5% in over at least 2 months of the pilot. ○ 84% of participants rated the intervention as helpful. ○ There was a significant improvement between the number of participants in full remission at baseline. ○ There was a significant improvement to interviewer-rated depression scores (MADRS; *p* = 0.014).
[63]	104 participants, aged 18–25 yy, with moderate depression symptomatology (DASS-21) and use of alcohol at hazardous levels (AUDIT)	The sample was randomly allocated to the DEAL Project or Web-based attention-control condition. The trial consisted of a 4-week intervention phase, including four 1-h modules in the areas of CBT, MI, BA, psychoeducation, relaxing, mindfulness and coping strategies, with follow-up assessment at post-treatment and at 3 and 6 months post-baseline. The outcomes were evaluated by PHQ-9 and TOT-AL.	DEAL Project it could be a good option for patients with both depression symptoms and alcohol use.	○ The DEAl Project was associated with statistically significant improvement in depression symptom severity (*d* = 0.71) and reductions in alcohol use quantity (*d* = 0.99) and frequency (*d* = 0.76) in the short term compared to the control group. ○ At 6-month follow-up, the improvements in the intervention group were maintained, although the difference between the interventions and control groups were no longer statistically significant.
[64]	257 Chinese adolescents, aged 13–17 yy, with mild-to-moderate depressive symptoms were recruited from three secondary schools in Hong Kong	The participants were randomly assigned to receive either intervention or attention control. The intervention is based on Grasping the Opportunity, a Chinese modified internet version of CATCH-IT. It is an internet program with 10 modules and includes monthly reminders by phone call or by messages through social media. The outcomes were evaluated by CESD-R at the 12-month follow-up.	Poor completion rate is the major challenge in the study.	○ Only 10% of the participants completed the intervention. ○ Compared to the attention control, Grasp the Opportunity led to reductions in depressive symptoms at the 12-month follow-up.
[65]	208 Dutch female adolescents with elevated depressive symptoms (RADS-2)	The sample was randomly allocated to one of four conditions: OVK program, SPARX program, OVK and SPARX combined and a monitoring control condition. Girls in the OVK condition were given eight lessons of OVK program by a professional psychologist at school, approximately 1-h per week. The SPARX condition consisted of weekly game play of SPARX, a fantasy game, at home, at the time of their choosing, asking to complete one level (20–40 min) per week. Depressive symptoms were measured at pre-test, weekly, at post-test, and at 3–6 and 12-month follow-up.	Videogames could be a good strategy to improve the compliance of adolescents for computerized CBT.	○ Depressive symptoms decrease in all conditions. ○ There is a maintenance in the reduction of depressive symptomatology at the 12-month follow-up.
[66]	107 participants (M 8%, F 92%), aged 17–48 yy, recruited at The University of Queensland Health Service	The sample was randomly assigned to LI-CBT arm versus self-help control arm. Outcomes were evaluated by K10, DASS-21, CSE, University Connectedness Scale, AUDIT.	It could be useful to introduce LI-CBT in the university system, even if further studies are needed.	○ Only 11% of distressed students agreed to join the treatment, and only 58% of LI-CBT participants attended any sessions. ○ The study arm showed a significant improvement in depression and anxiety at 2 months and over 12-month follow-up.
[67]	206 female students, aged 18–25 yy, at very high risk for eating disorder onset (WCS)	Women were randomized to IaM, a 10-week online preventive intervention including CBT, IPT, BA, stress management and problem solving or WLC. Assessments included EDI-2, EDE-Q, SCID and BDI-II.	IaM is an inexpensive, easy intervention that can reduce ED onset in high-risk women.	○ ED attitudes and behaviours improved more in the intervention than control group (*p* = 0.02). ○ For the 27 individuals with depression at baseline. Depressive symptomatology improved more in the intervention than control group (*p* = 0.016).
[68]	Web-based awareness and self-management protocol to mild-to-moderate depression	iFightDepression tool was based on cognitive behavioural therapy and addressed behavioural activation (monitoring and planning daily activities), cognitive restructuring (identifying and challenging unhelpful thoughts), sleep regulation, mood monitoring and healthy lifestyle habits. The tool is accompanied by a 3-h training intervention for health care professionals.	Protocol for the development, implementation and evaluation of the iFight Depression tool, cost-free, multilingual, guided, self-management program for mild-to-moderate depression cases.	○ Findings should be still collected, being a protocol.
[69]	927 students, enrolled in universities in Massachusetts, were recruited to join the web-based screening survey for depression.	Participants who screened positive, thorough PHQ-9, received a direct link to an online depression toolkit (including two websites, psychoeducational materials, information on a local suicide prevention helpline). Participants who screened positive for MDD or suicidality were given an opportunity to schedule a Skype consultation with a psychiatrist.	Current online technologies can provide depression screening and psychiatric consultation to college students.	○ 285 students screened positive of the 972 total students. ○ 76.4% found the interview useful in helping them understand their depression. ○ 88.2% thought that psychologists and psychiatrists could successfully see patients via VC.
**Obsessive-Compulsive Disorder**				
[70]	31 youth (19 M; 12 F), aged 7–16 yy, with OCD (CY-BOCS & ADIS-C/P)	The sample was randomly assigned to W-CBT or WLC. The online program consists of 14 sessions of family-based CBT over 12 weeks.	This preliminary study suggests the possible role of W-CBT in reducing OC symptoms in youth with OCD.	○ 81% of youth in the W-CBT arm were treatment responders, versus only 13% individuals in the WLC. ○ 56% of the individuals in the W-CBT group met remission criteria, versus only 13% in the WLC group.
[71]	22 child (13 M; 9 F), aged 4–8 yy, with OCD (ADIS-C/P & CY-BOCS)	The VTC-FB-CBT consists of 14 weeks of treatment through the VTC platform which uses computer games to enhance children’s understanding of treatment concepts. An 8-item and 36-item assessment were used to evaluate satisfaction and therapeutic alliance.	VTC methods may offer solutions to overcoming traditional barriers to care for early-onset OCD.	○ At post treatment, 72.7% on Internet cases and 60% of Clinic cases showed “excellent response”. ○ At follow-up 80% of Internet cases and 66.7% of Clinical cases showed “excellent response”. ○ VTC-FB-CBT showed strong engagement and satisfaction verified through the questionnaire.
[72]	3 female patients with a story of OCD	The patients were randomly assigned to a 1, 2 or 3-week baseline period prior to beginning the 12-week manualized CBT intervention. Sessions were delivered via VC, once a week and all sessions were 60 min in length. ERP was the primary intervention method. The assessment was made by SCID-I/P, Y-BOSC, CGI, WSAS, HDRS, WAI.	Manualized CBT for OCD can be effectively delivered via a VC network.	All three patients complete the entire 12 weeks of treatment. Substantial decrease in total Y-BOCS scores were observed. Follow-up ratings support the durability of VC-CBT for OCD.
[73]	6 patients (M 1; F 5) with history of OCD (ADIS)	The sample received 15 sessions of therapy delivered only over teleconference (six sessions) and cell phones (nine sessions) over a 3-month period. ERP was the primary intervention method.	Internet-delivery CBT may be a promise method treatment for OCD patients.	○ 4 patients, at the end of the therapy, were highly improved and no longer met diagnostic criteria for OCD according to ADIS and Y-BOCS. ○ The same was true at 3-month follow up although some small increases in OCD symptoms had occurred.
[74]	15 adults (M 13.3%; F 86.7%) with OCD	Patients were treated with a VC-mediated, twice weekly, ERP for adults. Assessments consist of SCID-IV, Y-BOCS, CGI, QLESQ, RTQ, WAI-S, CSS, TVS, PEAS.	This study adds to the growing body of literature suggesting that videoconference-based interventions are viable alternatives to face-to-face treatment.	○ All participants had improvements in OCD symptoms. ○ 10 individuals completed a 3-month follow-up assessment and 30% of participants no longer met the criteria for OCD. ○ 0% of participants were rated as very much or much improved in the CGI.
[75]	21 participants, aged 12–17 yy, with OCD (MINI-KID) and their parents	The intervention consists of a 12-week, 12 chapters, ICBT treatment delivered through film, exercises, animation, psychoeducational tools and interactive scripts. The outcomes measured through CY-BOCS, ChOCI-R, COIS-R, CGAS, SCAS C/P, CDI-s, SDQ, FAS-PR.	ICBT could be efficacious, acceptable and cost-effective for adolescents with OCD.	○ Treatment yielded significant improvements on all clinician-parent and most self-administered outcome measures. ○ At 6-month follow-up, 71% were classified as responders and 76% as being in remission.
[76]	72 adolescents, aged 11–18 yy, with OCD and their parents	The sample was randomized to receive specialist TCBT or face-to-face CBT. All participants received up to 14 sessions of CBT.	TCBT is an effective treatment and is not inferior to standard clinic-based CBT.	○ TCBT was not inferior to face-to-face CBT at post-treatment, 3 month, and 6-month follow-up. ○ At 12-month follow-up there were no significant between-group differences on the CY-BOCS. ○ Improvements made during treatment were maintained through to 12-month follow-up. ○ Participants in each condition report high levels of satisfaction.
[77]	30 children, aged 7-17, with primary diagnosis of OCD, and their parents	An open trial where all participants receive a newly developed enhanced CBT (eCBT), that include video conferencing sessions (supervision and guided exposure exercises at home = in addition to face-to face sessions; an app system of interconnected apps for the child, the parents and the therapist; psychoeducational videos; and frequent online self-assessments with direct feedback to patients and the therapist. Assessments are conducted pre-treatment, post-treatment, and at 3- 6- and 12- month follow-up.	NA	○ The findings are still ongoing.

CBT: Cognitive Behavioural Therapy; ICBT: internet based CBT; TCBT: Telephone CBT; W-CBT: web-camera CBT; VC: Videoconferencing; VTC: Video Teleconferencing; F: Female; M: Male; NET: Internet-based CBT; WLC: Waitlist control; CCAL: Camp Cope-A-Lot; TMH: Telemental Health; IPT: Interpersonal Psychotherapy techniques; BAC: Behavioral Activation; FCBT: family based CBT; VTC-FB-CBT: Video Teleconferencing-delivery family-based cognitive-behavioral therapy; CBM-A: Cognitive Bias Modification of Attention; ABM: Attentional Bias Modification; CBM-I: Cognitive Bias Modification for Interpretations; CATCH-IT: Competent Adulthood Transition with Cognitive-behavioral Humanistic and Interpersonal Training; OAPA: Online Assessment of Preschool Anxiety; ADIS-C/P: Anxiety Disorder Interview Schedule: Child and Parent; W-SFBT: Web-Solution Focused Brief Therapy DASS-21: Depression and Anxiety Stress Scale-21 CY-BOCS: Children’s Yale-Brown Obsessive Compulsive Scale; K-SADS-P: Affective Disorders and Schizophrenia for School Age Children-Present Episode; SPSQ-C: Social Phobia Screening Questionnaire for Children up to 18 Years Old; MADRS-S: Montgomery-Åsberg Depression Rating Scale; SCAS-C/P: The Spence Children’s Anxiety Scale-Child/Parent Version CDI: Children’s Depression Inventory; yy: years; OC: Obsessive Compulsive; OCD: Obsessive-compulsive Disorder; BTPS: Barriers to Treatment Participation Scale; WAI: Working Alliance Inventory, Parent-Report and Therapist-Report; CSQ-8: Client Satisfaction Questionnaire; SCARED: Screen for Child Anxiety Related Emotional Disorders; CGI: Clinical Global Impressions Scale; MASC-C/P: Multidimensional Anxiety Scale for Children, Child and Parent Reports; CBCL: Child Behavior Checklist; FACLIS: Family Accommodation Checklist and Interference Scale; BAI: Beck’s Anxiety Inventory; CCAPS College Counseling Assessment of Psychological Symptoms; Therapist-assisted Online Parenting Strategies; BDI: Beck Depression Inventory; CIS-R: Revised Clinical Interview Schedule; QUALYs: quality-adjusted life-years; CES-D: Center for Epidemiologic Studies Depression Scale; HADS: Anxiety subscale of the Hospital Anxiety and Depression Scale; PSWQ: Penn State Worry Questionnaire PHQ-A: Patient health Questionnaire-9 modified for Adolescent; CASQ-R: The Revised Children’s Attributional Style Questionnaire; RSE: Rosenberg Self-Esteem Scale; ADHEALTH: National Longitudinal Study of Adolescent Health; CDSR-R: Children’s Depression Rating Scale Revised; HAMD: Hamilton Depression Rating Scale for Depression; MINI: Mini International Neuropsychiatric Interview; DEAL: Depression Alcohol Project; AUDIT: Alcohol Use Disorders Identification Test; RADS-2: Reynolds Adolescent Depression Scale; OVK: school-based CBT prevention program ‘OP Volle Kracht’; SPARX program: computerized CBT program “SPARX”; LI-CBT: Low-Intensity cognitive-behaviour therapy; K10: Kessler10; CSE: Coping Self-Efficacy scale; WHO5: 5-item World Health Organization Well-Being Index; IaM: Image and Mood program; WCS: Weight Concerns Scale; ED: Eating Disorder; EDE-Q: Eating Disorder Examination Questionnaire; EDI-2: Eating Disorder Inventory BDI-II: Beck Depression Inventory-II; ERP: Exposure and Response Prevention; SCID: Structured Clinical Interview for the DSM; Y-BOSC: Yale-Brown Obsessive Compulsive Scale; WSAS: Work and Social Adjustment Scale; WAI-S: Working Alliance Inventory; MINI-kid: Mini-International Neuropsychiatric Interview for Children and Adolescents; QLESQ: Quality of Life Enjoyment and Satisfaction Questionnaire short form; RTQ: Reaction to Treatment Questionnaire; CSS: Client Satisfaction Survey; TVS: Telepresence in Videoconference Scale; PEAS: Patient EX/RP Adherence Scale; ChOCI-R: Children’s Obsessional Compulsive Inventory Revised; COIS-R: Child Obsessive-Compulsive Impact Scale; CGAS: Children’s Global Assessment Scale; SDQ: Strengths and Difficulties Questionnaire; FAS-PR:: Family Accommodation Scale, Parent-Report.

**Table 3:** Core competencies and skills in TMH by Adolescent Mental Health professional.

**Basic technical/IT skills**	Informatics, notebook, desktop, online videoconferencing, online platforms for offering TMH services, etc. These competencies include as well know and explain to the patients how to connect their devices to the TMH platform, and many other potentially occurring technical issues.
**Assessment skills in TMH**	When a clinician plans to implement an adolescent TMH service, an assessment should be proposed, also for stratifying patients who need a TMH vs. face-to-face mental health care.
**Relational skills in TMH**	Relational skills in digital world may differ from that in-person, particularly with adolescents. Understanding and learning how to build a good relationship and therapeutic alliance with the youngsters during TMH sessions. A good way to build a strong and collaborative alliance and interaction may consist in involving the adolescent (and their family, if needed) also in the technical process of setting up TMH session from the beginning.
**Communication skills in TMH**	Understanding and learning how to communicate via TMH with adolescents (‘how to do’, ‘how not to do’). An adequate training on how to properly use nonverbal communication may play a crucial role in building an authentic experience through expression of empathy, professionalism, and therapeutic intentions. In particular, these communication skills are essential with youngsters, by guaranteeing a good eye contact, using an exaggerated tone of voice, a variegated range of facial expressions, and energetic hand gestures. Clinicians should benefit in previously practicing nonverbal gestures, including facial expressions on camera in advance (before the session) in order to ensure that those are visible in the camera frame during TMH session.
**Collaborative and inter-professional skills in TMH**	How to collaborate and share professional insights and viewpoints in describing a clinical case, supervision and in providing e-consultations in TMH with other colleagues.
**Administrative skills in TMH**	How to track TMH intervention (i.e., electronic and/or paper medical diary), prescription (i.e., electronic and/or paper prescription delivered by mail, email or through general practioner), how to manage payments and receipts, etc.
**Medico-legal competencies** **and skills in TMH**	Regulatory and ethical issues and how to manage them, with a particular focus on adolescent psychiatry, how to create and obtain an ad hoc informed consent, how to create and obtain an ad hoc plan emergency shared plan; privacy and safety issues, responsibility during a TMH session, with a particular focus on adolescent psychiatry, etc.
**Ethno- and cultural psychiatry skills in TMH**	Adequately verify and investigate the culturally and religiously determined resistances in offering a TMH session according to the culture and religion of patients, particularly in the case of children and adolescent patients.

**Table 4:** Before starting.

**General knowledge and experience about TMH**	Clinicians should ask whether the patient (and their parents/caregiver) has even seen a physician through telemedicine (or TMH) and, if not, whether he/she has used any online videoconferencing. If the patient (and their parents/caregiver) has not seen a physician through an online videoconferencing, it could be helpful to make references to common lay technology, such as telephony, Facetime^®^, Skype^®^ and so on, and explain key differences of these online videoconferencing platforms.
**General notions about TMH**	Clinicians could briefly provide some historical background on why TMH has been firstly used (i.e., to bring the patient specialty care without burden to the patient of travel or missing work).
**Specific notions about the efficacy and effectiveness of TMH interventions**	Clinicians could briefly provide some evidence-based data on studies about the application of TMH amongst adults and youngsters as well as cite some relevant data on the use of TMH intervention in specific mental disorders.
**Explanation on** **how TMH works**	Clinicians should explain to young patients (and their parents/caregiver) that the TMH session may happen in a “real time” (i.e., synchronous) or asynchronous modality; explain key differences between two modalities and when we recommend asynchronous versus synchronous modality in providing a TMH intervention.
**Clarification about** **recording TMH session**	Clinicians should inform young patients (and their parents/caregiver) whether or not a TMH session will be recorded. If a clinician desires to record a TMH session, then he/she must obtain an explicit and written consent from the adolescent patient and his/her parents/caregiver. If a clinician desires to record a TMH session, then he/she should provide motivations and explanations about this choice.
**Establishing a visual context (i.e., setting) of TMH session**	For youngsters, clinicians should be aware that providing the possibility of a virtual tour of their office might assure young people that none else is present and/or give some context to the clinical setting in which TMH session is taking place. In some settings, it may be possible or even desirable, to allow patients to manipulate the remote control during the TMH session. Clinicians should clearly state which is the setting in which the TMH session is going (i.e., inpatient service, traditional outpatient service, public versus private practice, university hospital with the possibility that students may assist the TMH session, nonmedical facilities such as schools, juvenile justice settings, primary care, or nonclinical settings such as home). The type of service site will have implications for the model of care and operational procedures, such as staffing, patient selection, patient management, safety, and emergency planning.
**Discussing how to manage occurring technical issues**	Clinicians are encouraged to discuss any technical difficulties and how to manage them (i.e., slight lag in audio, video or distortions, lack of Wi-Fi connection, etc.). Clinicians should firstly prepare and provide to patient a B plan in case any technical issue may occur and interfere with TMH session. Adolescents with a parent or other family member present may need a camera with an appropriate adjustment to accommodate two or more individuals in the frame.
**Offering a space** **for open questions**	Clinicians should give the possibility to young patients (and their parents/family) to ask questions before starting TMH session. This may be especially helpful to younger patients (and their parents/caregiver) who are not as comfortable with electronic media.
**Obtain informed consent**	Clinicians should evaluate if they prefer to obtain informed written and signed consent in-person (maybe during a first pre-evaluation visit) or electronically (i.e., by email). Clinicians should evaluate if they prefer to perform an ad hoc informed consent for TMH different from that used to provide in-person intervention.
**Obtain written and signed emergency** **shared plan**	A key component of TMH care delivery is developing a comprehensive safety and emergency management planning including standard operating procedures and protocols for managing urgent needs and psychiatric emergencies, including a concrete crisis plan with the patient and the family. Clinicians should agree with young patient (and their parents/caregiver) who is the referee person in case of any emergency occurring during a TMH session and which is the plan to be followed in any case. An emergency plan should clearly assign responsibility for contacting emergency and other necessary personnel in the event of an emergency. Clinicians shall consider involving family members in emergency treatment situations when possible and clinically appropriate, particularly in case of young patients. Clinicians should be aware and know if specific emergency procedures exist for any site (e.g., school) where the TMH session takes place. If none exists, clinicians should establish emergency procedures, including who will do what at each site to ensure coordination, a prompt management and intervention in emergencies. Emergencies occurring between visits should be managed as for usual.

**Figure A1:**
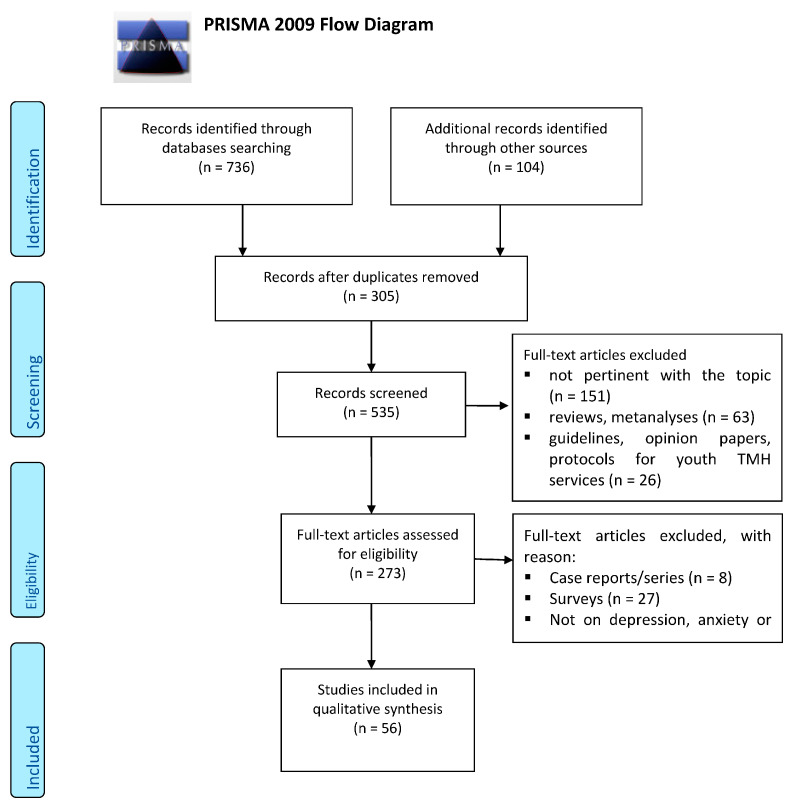
PRISMA 2009 Flow Diagram.

## Data Availability

Not applicable.

## Appendix A

**Table A1:** MEDLINE search strategy.

SET	MEDLINE
1	Telepsychiatry
2	Telemental Health
3	Telepsychotherapy
4	Videoconferencing
5	Tele *
6	Remote
7	Sets 1–6 were combined with “OR”
8	Youth Mental Health
9	Depress *
10	Anxiety
11	Obsessive Compulsive
12	Sets 8–11 were combined with “OR”
13	*Telemental health*
14	*Telepsychiatry*
15	*Adolescent Psychiatry*
16	Sets 13–15 were combined with “OR”
17	Sets 7, 12 and 16 were combined with “AND”
18	Set 17 was limited to 25 January 2021
	Humans, no language or time restriction

Words written in *italic* were used as MeSH headings, the others were used as free text. *, the search strategy chosen in the pubmed.
